# 1951 Influenza Epidemic, England and Wales, Canada, and the United States

**DOI:** 10.3201/eid1204.050695

**Published:** 2006-04

**Authors:** Cécile Viboud, Theresa Tam, Douglas Fleming, Mark A Miller, Lone Simonsen

**Affiliations:** * National Institutes of Health, Bethesda, Maryland, USA;; † Public Health Agency of Canada, Ottawa, Ontario, Canada;; ‡ Royal College of General Practitioners, Harborne, Birmingham, United Kingdom

**Keywords:** influenza, pneumonia, excess mortality, pandemic, epidemic, United States, England, Canada, viral strains, immunity, transmissibility, research

## Abstract

Influenza poses a continuing public health threat in epidemic and pandemic seasons. The 1951 influenza epidemic (A/H1N1) caused an unusually high death toll in England; in particular, weekly deaths in Liverpool even surpassed those of the 1918 pandemic. We further quantified the death rate of the 1951 epidemic in 3 countries. In England and Canada, we found that excess death rates from pneumonia and influenza and all causes were substantially higher for the 1951 epidemic than for the 1957 and 1968 pandemics (by >50%). The age-specific pattern of deaths in 1951 was consistent with that of other interpandemic seasons; no age shift to younger age groups, reminiscent of pandemics, occurred in the death rate. In contrast to England and Canada, the 1951 epidemic was not particularly severe in the United States. Why this epidemic was so severe in some areas but not others remains unknown and highlights major gaps in our understanding of interpandemic influenza.

## Abstract 2

Death rates were substantially higher for England and Canada than for the United States.

Influenza is responsible for large increases in deaths in pandemic seasons when emerging viral subtypes with novel surface antigens become predominant, and also in some interpandemic seasons, when established subtypes exhibit antigenic drift (*1*). The circulating viral subtype is associated with varying severity of influenza epidemics (*2*): in the last 2 decades in the United States, estimated excess death rates were on average 2.8-fold higher in A/H3N2-dominated seasons than in A/H1N1 and B seasons (*3*). Within a given subtype, however, the strain-specific determinants of epidemic severity are still poorly understood. For instance in the United States in the same period, excess death rates varied nearly 4-fold among A/H3N2 seasons, even after adjustments for population aging (*3*). Better characterizations of past severe influenza epidemics can help understand and perhaps help predict the occurrence of severe epidemics.

Anecdotal accounts exist in the literature of historical influenza epidemics associated with unusual numbers of deaths, such as occurred in the 1951 epidemic in England in the midst of the first era of A/H1N1 viruses (1918–1957) (*4*). In Liverpool, where the epidemic was said to originate, it was "the cause of the highest weekly death toll, apart from aerial bombardment, in the city's vital statistics records, since the great cholera epidemic of 1849" (*5*). This weekly death toll even surpassed that of the 1918 influenza pandemic (Figure 1).

**Figure 1:**
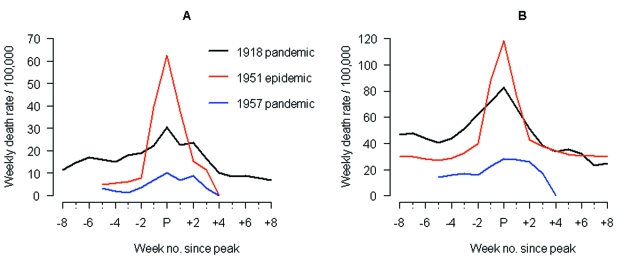
Comparison of 1951 epidemic (A/H1N1) with the 1918 and 1957 pandemics (A/H1N1 and A/H2N2, respectively) in Liverpool, England. Time series of weekly death rates from A) respiratory causes (pneumonia, influenza and bronchitis) and B) all causes. Epidemics were aligned at the week of peak mortality (peak week = week ended Feb 22, 1919; Jan 13, 1951; Oct 12, 1957). The 1918 pandemic occurred in 3 waves in Liverpool (summer 1918, autumn 1918, winter 1919); the "third wave" was associated with the highest death rate and is represented here.

The international pattern of influenza-related deaths in 1951 has not been adequately quantified in the past because of lack of methodologic tools and historical death records. However, this historical epidemic is a good example to illustrate major gaps in our current understanding of influenza virus epidemiology. We revisited the 1951 epidemic by quantifying its death rate in 3 countries (England and Wales, Canada, the United States) and comparing its age-specific mortality pattern with that of surrounding epidemic and pandemic seasons (*1*).

## Methods

### Data

We obtained monthly pneumonia and influenza (P&I) and all-cause numbers of deaths for 1950 to 1999 from Health Canada (*6*), by 5-year age groups (details on the International Classification of Diseases codes used are given in Table 1). Canada was the only country with detailed age-specific mortality data for the 1950s readily available in electronic format.

**Table 1:** Codes from the International Classification of Diseases (ICD) used for selecting deaths from pneumonia and influenza (P&I) in Canada and England and Wales, 1950–1999

ICD revision	Canada	England & Wales	Codes used for P&I deaths
ICD-6	1950–1957	1950–1957	480–483; 490–493
ICD-7	1958–1968	1958–1967	480–483; 490–493
ICD-8	1969–1978	1968–1978	470–474; 480–486,
ICD-9	1979–1994	1979–1999	480–487
ICD-10	1995–1999		J10.0–J11.8; J12.0–J18.9

**Figure 2:**
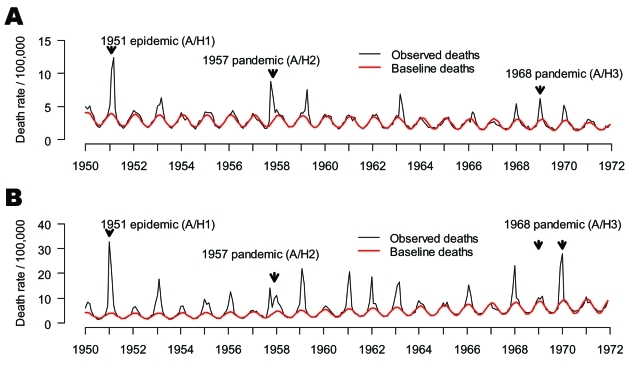
Time series of monthly mortality from pneumonia and influenza (P&I, represented as death rate/100,000) from 1950 to 1972 in A) Canada and B) England and Wales. Black line: observed deaths, Red line: baseline deaths predicted by a seasonal regression model. Note the 2 arrows for the 1968 pandemic in England, representing the 2 waves of the smoldering A/H3N2 pandemic (1968–69 and 1969–70, respectively) (*18*).

For England and Wales (referred to as "England" for simplicity), we compiled P&I and all-cause deaths by month for 1950 to 1999 from the Registrar General (1950–1958 [7], ), and National Statistics (1959–1999 [8], ). In both countries, monthly deaths were normalized by population size to obtain comparable death rates over time and these were standardized to 30.5-day months (Figure 2). Population data were obtained from the same agencies (*6**,**8*).

As US monthly vital statistics were not available electronically since 1950, we compiled excess death estimates from various historical publications (*9**–**12*). These estimates were based on National Vital Statistics and death records from P&I and all causes in major American cities compiled by the Centers for Disease Control and Prevention and derived from excess mortality models similar to ours (see below).

We also conducted a literature search to compile reports describing the local patterns and geographic spread of the 1951 influenza epidemic in the 3 countries (*5**,**9**,**13**,**14*). Moreover, we obtained mortality data specifically for Liverpool, where the 1951 epidemic had the highest impact and death records have been previously described (*5**,**13**,**15**,**16*) (Figure 1).

### Seasonal Excess Death Rate Estimates, Canada and England

Our primary goal in this study was to compare the death rate of the 1951 epidemic with that of the 1957 and 1968 pandemics. For this purpose, we fit a seasonal model to P&I and all-cause deaths for 1950 to 1971, capturing all 3 influenza seasons of interest, as described below. We present monthly time series and seasonal estimates for this period (20 seasons, see Figure 2 for P&I). A secondary goal was to compare the age mortality pattern of the 1951 epidemic with that of other influenza seasons. To have more statistical power and analyze several influenza seasons with substantial death rates, we also used an extended study period, 1950–1999.

For Canada and England, we applied a modified version of Serfling's classical seasonal regression model to monthly data on death rates for each country (*17*), as described elsewhere (*3**,**18*). We obtained a baseline for deaths in the absence of influenza, separately for each outcome (P&I and all-cause) and available age group (see Figure 2 for P&I). Seasonal excess deaths were then estimated as the number of deaths in excess of the baseline during months of increased influenza activity.

### Standardization of Seasonal Excess Death Rates

Since our goal was to compare influenza deaths across multiple seasons and countries, we had to control for baseline differences in demography, healthcare, and socioeconomic status that may affect influenza-related deaths. To this end, we calculated age-adjusted seasonal excess death estimates in a manner previously described (*3**,**18*). Further, to control for residual differences in baseline death rates related to health and socioeconomic status, we adjusted the seasonal estimates for temporal changes in mortality in the summer months, when influenza is absent (*18*). We used year 1960, midpoint of the main study period 1950–1971, as an index.

### Age-Specific Patterns of Seasonal Excess Death Rates, Canada

We examined whether the 1951 epidemic had an epidemic or pandemic mortality age pattern, as indicated by a shift in the age distribution of deaths towards younger age groups (*1*). In Canada, the 1950–51 season was the first season in our mortality records with complete age details. Hence, we could not evaluate a potential age shift between earlier seasons and the 1950–51 season, as described elsewhere (*1*).

We therefore developed an alternative method to identify a pandemic signature, in which we compared the gradual increase of influenza-related deaths with age between epidemic and pandemic seasons. We first used all moderate-to-severe influenza seasons in the interpandemic periods to obtain a null distribution of mortality age patterns during epidemics (we chose the 17 seasons above the median). Second, we checked that we could actually detect a pandemic age pattern by comparing the null epidemic pattern with those of the 1957 and 1968 pandemics. Then, we compared the null pattern with that of the 1951 epidemic. To model the gradual increase of influenza-related deaths with age in adults, we fitted an exponential to unadjusted P&I excess death rates by 5-year age groups for persons >55 years of age. The test then relied on comparing between seasons the values of the age and intercept coefficients of the exponential models. Bootstrap resampling of influenza seasons in the interpandemic periods yielded a p value for the test.

## Results

### Geographic and Temporal Spread

Influenza activity started to increase in Liverpool, England, in late December 1950 (*5**,**13*). The weekly death rate reached a peak in mid-January 1951 that was ≈40% higher than the peak of the 1918–19 pandemic, reflecting a rapid and unprecedented increase in deaths, which lasted for ≈5 weeks [5] and Figure 1). Since the early 20th century, the geographic spread of influenza could be followed across England from the weekly influenza mortality statistics in the country's largest cities, which represented half of the British population (*13*). During January 1951, the epidemic spread within 2 to 3 weeks from Liverpool throughout the rest of the country.

For Canada, the first report of influenza illness came the third week of January from Grand Falls, Newfoundland (*19*). Within a week, the epidemic had reached the eastern provinces, and influenza subsequently spread rapidly westward (*19*).

For the United States, substantial increases in influenza illness and excess deaths were reported in New England from February to April 1951, at a level unprecedented since the severe 1943-44 influenza season. Much milder epidemics occurred later in the spring elsewhere in the country (*9*).

Local disparities were found in all 3 countries, with a consistent pattern of higher numbers of deaths in locations affected earlier (*9**,**13**,**14*). In England, influenza-related death rates were ≈3-fold higher in Liverpool than in the rest of the country (*13*). In Canada, death rates were ≈2.4-fold higher in the eastern seaboard provinces than in the rest of the country (*13**,**14*). Similarly, in the United States, rates were ≈2.3-fold higher in New England than in the rest of the country (*9*).

### Patterns of Seasonal Excess Death Rates, All Ages, 3 Countries

**Figure 3:**
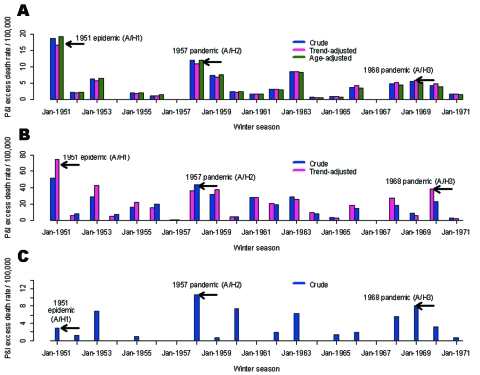
Seasonal pneumonia and influenza (P&I) excess death rates, all ages, 1950–51 to 1970–71. Crude death rates (blue bars), death rates adjusted for summer trends in mortality unrelated to influenza (pink bars) and adjusted for demographics (green bars). A) Canada. B) England and Wales. C) USA. Note that for England, comparisons used the second A/H3N2 pandemic wave of major impact (*18*).

Crude and adjusted seasonal excess death estimates in the 3 countries are presented for the period 1950–1971 in Figure 3 (P&I). A specific comparison of the 1951 epidemic and 1957 pandemic is provided in Table 2 (P&I and all-cause).

**Table 2:** Comparison of influenza-related death rates in the 1951 influenza epidemic (A/H1N1) and 1957 influenza pandemic (A/H2N2), Canada, England and Wales, and United States*

Setting	Age group	P&I† excess death rate/100,000 (RR‡)	All cause excess death rate/100,000 (RR‡)
Canada (13.7 million)§			
	1951 epidemic (Jan –Apr)	All ages	18.6 (1.54)	34.1 (1.47)
		<65 y	6.6 (0.90)	14.8 (1.09)
		>65 y	164 (2.41)	329 (2.22)
		1957 pandemic (Sep–Dec)	All ages	12.1 (1.00)	23.2 (1.00)
			<65 y	7.3 (1.00)	13.6 (1.00)
			>65 y	68.0 (1.00)	148 (1.00)
England & Wales (43.8 million)§			
	1951 epidemic (Jan –Mar)		All ages	50.1 (1.40)	178 (1.45)
	1957 pandemic (Oct 1957–Mar 1958)	All ages	35.8 (1.00)	123 (1.00)
United States (154.9 million)§			
	1951 epidemic (Feb–Apr)	All ages	5.5 (0.48)	9.0 (0.25)
	1957 pandemic (Oct 1957–Mar 1958)	All ages	11.5 (1.00)	36.3 (1.00)

*Unadjusted death rates (see Figure 3 for rates adjusted for demographics and trends in health care and socioeconomic status). †P&I, pneumonia and influenza. ‡RR, ratio of excess death rate in 1951 to excess death rate in 1957. §Population in 1951.

For Canada, the 1951 epidemic was the most severe influenza season in the period 1950–1999, as indicated by crude seasonal excess death rates from P&I and all causes (data not shown). On the basis of both outcomes, the 1951 epidemic caused a 1.5-fold higher death rate than the 1957 pandemic; the rate was 3- to 4-fold higher than that seen in the 1968 pandemic. Adjusting for factors unrelated to influenza, such as demographics, health, and socioeconomic status only marginally modified our estimates (by <11%).

In England, the 1951 epidemic had similar death patterns. It was responsible for the largest increase in winter deaths from P&I and all causes in the period 1950–1999 (data not shown), with 1.3- to 1.4-fold higher crude excess death rates than those seen in the 1957 and 1968 pandemics. In contrast to Canada, adjusting for trends in demographics and health care substantially changed our excess death estimates, exacerbating the impact of the 1951 epidemic. Baseline P&I summer death rates doubled from 1950 to 1970 (Figure 2), probably because of rapid aging of the British population. During these 2 decades, the proportion of persons >65 years of age increased by 2.3% in England, which explains the trend in British death rates; in comparison, it increased by only 0.3% in Canada (*6**,**8*).

In the United States, by contrast, the 1951 epidemic was not particularly severe, except possibly in the New England region, adjacent to Canada. In the United States, this epidemic ranked with low-to-moderate influenza seasons, with only half the impact of the 1957 pandemic for P&I deaths and even less for all-cause deaths.

Estimation of crude and adjusted excess deaths suggests that the 1951 epidemic was unusually severe in England and Canada but not in the United States. The absolute rates of excess deaths were very different between countries, with systematically higher rates in England (by 3- to 5-fold, Table 2). The difference remained even after adjusting for international differences in demographics and healthcare and was also found for deaths from all causes, which controls for potential differences in the coding of death certificates (Table 2). Such international discrepancies in influenza-related death rates have been highlighted on several occasions in the past, although not elucidated (*18**,**20**–**22*). Because of these unresolved differences, this analysis focused on the relative impact of the 1951 epidemic as compared with surrounding influenza seasons.

### Age-specific Patterns of Seasonal Excess Death Rates, Canada

Given the unusual death rate of the 1951 influenza epidemic in England and Canada, we hypothesized that an emerging virus subtype may have circulated there, perhaps with pandemic potential. We investigated the age-specific mortality pattern of the 1951 epidemic in Canada in relation to other seasons to address this aspect.

**Figure 4:**
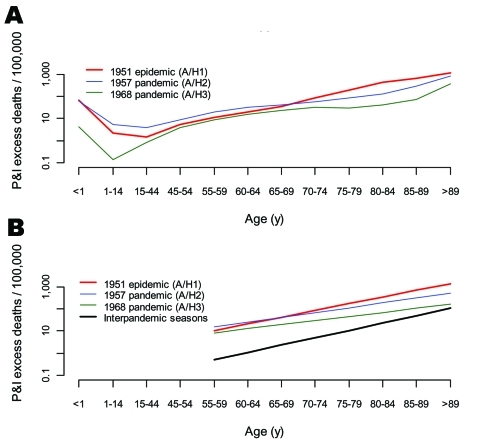
Age-specific pneumonia and influenza (P&I) excess death rates in the 1951 influenza epidemic, 1957, and 1968 pandemics, Canada. A) Observed. B) Exponential models using 5-year age groups starting at age 55 years and ending at >90 years (R^2^ >0.85 for all seasons). Black curve: "null distribution" of expected pattern in epidemic seasons, based on major epidemics in the interpandemic periods, 1950–1999 (N = 17). The age coefficient was set at the mean of the "null" distribution (see Table 3 for values). The intercept was set at the minimum of the distribution for legibility.

Inspection of P&I excess death rates by age shows that the 1951 epidemic had the typical pattern of death rates found in other epidemics, steeply increasing with age after infancy (Figure 4). Statistical analysis showed that the age pattern in 1951 was well within the range of the null distribution of reference epidemic seasons in influenza interpandemic periods (Table 3). Conversely, we found lower age coefficients for the 1957 and 1968 pandemics as compared with reference epidemic seasons, illustrating that deaths increased less with age in pandemics than in epidemics and that our statistical approach could detect a pandemic signature (p<0.001) (*1*).

**Table 3:** Test for a pandemic signature in age-specific, influenza-related death rates of 1951 influenza epidemic, Canada*

Influenza seasons	Age coefficient	Intercept	p value†
Major epidemic seasons, 1950–1999‡			
Average (SD)§	0.15 (0.02)	–7.9 (1.9)	Reference
Minimum-maximum	(0.11–0.18)	(–9.9; –4.1)	Reference
1951 epidemic (A/H1)	0.14	–5.2	0.30; 0.13
1957 pandemic (A/H2)	0.10	–2.7	<0.001¶
1968 pandemic (A/H3)	0.08	–2.5	<0.001¶

*Coefficients of exponential models explaining the increase in pneumonia and influenza (P&I) excess death rate with age for the 1951 epidemic, 1957 and 1968 pandemics, and other major epidemics in the interpandemic periods, 1950–1999. Exponential models use 5-year age groups starting at age 55 years and ending at age >90 years (see Figure 4). All *R*^2^ between 0.85 and 0.99 (*R*^2^ of linear model applied to log-transformed P&I excess death rate, equivalent to an exponential model for untransformed P&I). †p value for comparison of respectively age and intercept coefficients with the "null" distribution of major epidemic seasons. Based on 1,000 bootstrap samples. ‡"Null" distribution of major influenza seasons in the interpandemic periods, 1950–1999. Based on 17 seasons that had seasonal P&I excess mortality rates above the median of all seasons (including seasons in the A/H1N1, A/H2N2, and A/H3N2 era but excluding the 1951 epidemics and the 2 pandemics). §SD, standard deviation based on bootstrap resampling of the null distribution of observed epidemic patterns. ¶Evidence of pandemic signature (not consistent with an epidemic mortality age pattern).

## Discussion

We have shown that the 1951 influenza epidemic had greater death rate than all subsequent influenza epidemics or pandemics in England and Canada. In Canada, where age-detailed data were available, deaths in persons <65 years of age attributable to the 1951 epidemic were nearly equivalent to those of the 1957 pandemic. But what sets the 1951 epidemic apart from pandemics is that the older population was also severely affected, with twice the deaths as occurred in the 1957 pandemic. By contrast, the 1951 epidemic had minor impact in the United States, except possibly in New England.

To study influenza death patterns, we used P&I deaths, a reliable proxy for the timing and relative impact of influenza epidemics, as well as all-cause deaths, an indicator of their overall impact (*23*). Cardiovascular deaths are also widely used to quantify the impact of influenza (*24**,**25*); however, they were not available to us for this study. But since winter increases in P&I, cardiovascular, and all-cause deaths are synchronized and correlated in amplitude during influenza epidemics (*25*), we capture here the timing and death rate of these epidemics.

Influenza-related death rates reflect the combination of 2 underlying epidemiologic parameters: the attack rate, a measure of a pathogen's transmissibility, and the case-fatality rate, a measure of a pathogen's virulence. The unusual severity of the 1951 epidemic in England and Canada may stem from higher attack rates, higher case-fatality rates, or both. To isolate these factors, we examined Liverpool, England, where comparable data on illness and death exist for the 1951 and 1957 pandemics (*5*). In Liverpool, influenza attack rates in schoolchildren were 3-fold lower in 1951 than in 1957, which suggests lower transmissibility in this age group in 1951. By contrast, an equal number of influenza-related deaths occurred in the 2 seasons in children in Liverpool, which suggests a higher case-fatality rate in 1951 than in 1957. Similar findings were observed in the working adult population (*5*), an indication of unusual virulence in the influenza virus circulating in 1951 in Liverpool. This argument must be taken with caution, however, since most influenza-related deaths occur in the elderly (*3*), and attack rates are not available for this age group. Estimation of the transmissibility of the 1951 virus using a mathematical model for influenza transmission fitted to mortality data (*26*) might answer this question, not only in Liverpool but also in the rest of England and Canada.

Laboratory surveillance data from the World Health Organization (WHO) indicate that influenza A viruses circulating at the time were characterized as H1N1 (*27*), a subtype circulating since the 1918 pandemic (*28*). Although an unusually large drift event in the hemagglutinin of A/H1N1 viruses was reported in 1947 (*29*), subsequent changes in this protein remained minor until after 1951 (*27*). Hence, no virologic evidence of a shift or unusual drift in the hemagglutinin antigen exists for 1951 viruses. In support of the virologic evidence, we have shown that no epidemiologic pandemic signature occurred in 1951, as indicated by an age shift of deaths towards younger age groups (*1*).

The 1951 epidemic exhibited geographic disparities in influenza-related deaths, as illustrated by the contrast between England and Canada (countries with high death rate) and the United States (low death rate). These disparities are in part explained by laboratory surveillance reports by WHO (*27**,**30*), indicating that 2 antigenically distinct influenza A/H1N1 strains cocirculated in the Northern Hemisphere during the 1951 epidemic (*27**,**30*). The so-called "Scandinavian strain" was isolated in northern Europe and associated with mild illnesses. By contrast, the "Liverpool strain" was associated with severe illnesses and high deaths in Great Britain, Canada, southern Europe, and Mediterranean countries (*27*). As both strains cocirculated in some countries (*27*), intrasubtypic cross-immunity might have existed, with these 2 strains competing for susceptible hosts.

The precise reasons for the unusually high death rate associated with the Liverpool strain remain elusive. The genetic markers of influenza virulence are still unclear today, but a multibasic cleavage site in the hemagglutinin, as well as minor changes in internal genes, are believed to enhance viral pathogenicity (*31**,**32*). Only hemagglutinin inhibition tests could be performed in 1951, and to our knowledge, no influenza virus isolate or genetic sequence from 1951 is available in the public domain. Further molecular analysis of 1951 influenza specimens could help explain the extreme local pathogenicity in that season.

We have described an influenza season that was unexpectedly severe in some countries and mild in others. This geographic disparity in influenza-related deaths is not common; influenza mortality is generally correlated between the United States and Europe and within the United States (*33**,**34*). Occasional disparities have been reported, however. For instance, the impact of the 2 waves of the 1968 pandemic differed markedly between North American and Eurasian countries, perhaps because of differences in preexisting immunity and evolving viruses (*18*). In this context, the 1951 epidemic appears as another striking example of geographic disparities in influenza impact, perhaps explained in this case by cocirculation of 2 influenza A/H1N1 strains. Other competing hypotheses include differences in preexisting population immunity or socioeconomic factors, but these are less parsimonious explanations.

Many countries are actively preparing for the next influenza pandemic (*35**–**37*). Previous pandemics in the 20th century have been responsible for large numbers of deaths in all age groups (*1*); however, the age pattern of deaths in the 1918 and 1968 pandemics suggest that the elderly may actually be relatively protected against an emerging pandemic virus (*35**,**38**,**39*). By contrast, we have shown that the 1951 epidemic was not associated with the emergence of a new influenza subtype, yet had a higher death rate than 2 of the 3 pandemics of the past century in England and Canada, especially among the elderly, and higher death rate than all 3 pandemics in Liverpool. We conclude that pandemics are not always more severe in terms of deaths than epidemics, for reasons still unclear. A thorough investigation of the full genome of the influenza viruses involved in the unusually severe 1951 epidemic could shed light on the virulence and transmissibility factors at play and fill key gaps in our current understanding of interpandemic influenza (*40*).
